# The expression of delta opioid receptor mRNA in adult male zebra finches (*Taenopygia guttata*)

**DOI:** 10.1371/journal.pone.0256599

**Published:** 2021-08-31

**Authors:** Pooja Parishar, Neha Sehgal, Soumya Iyengar

**Affiliations:** National Brain Research Centre, Gurugram, Haryana, India; Texas Christian University, UNITED STATES

## Abstract

The endogenous opioid system is evolutionarily conserved across reptiles, birds and mammals and is known to modulate varied brain functions such as learning, memory, cognition and reward. To date, most of the behavioral and anatomical studies in songbirds have mainly focused on μ-opioid receptors (ORs). Expression patterns of δ-ORs in zebra finches, a well-studied species of songbird have not yet been reported, possibly due to the high sequence similarity amongst different opioid receptors. In the present study, a specific riboprobe against the δ-OR mRNA was used to perform fluorescence in situ hybridization (FISH) on sections from the male zebra finch brain. We found that δ-OR mRNA was expressed in different parts of the pallium, basal ganglia, cerebellum and the hippocampus. Amongst the song control and auditory nuclei, HVC (abbreviation used as a formal name) and NIf (nucleus interfacialis nidopallii) strongly express δ-OR mRNA and stand out from the surrounding nidopallium. Whereas the expression of δ-OR mRNA is moderate in LMAN (lateral magnocellular nucleus of the anterior nidopallium), it is low in the MSt (medial striatum), Area X, DLM (dorsolateral nucleus of the medial thalamus), RA (robust nucleus of the arcopallium) of the song control circuit and Field L, Ov (nucleus ovoidalis) and MLd (nucleus mesencephalicus lateralis, pars dorsalis) of the auditory pathway. Our results suggest that δ-ORs may be involved in modulating singing, song learning as well as spatial learning in zebra finches.

## Introduction

The endogenous opioid system consists of the opioid ligands endorphin, enkephalin and dynorphin and the receptors that they preferentially bind to, that is, the μ, δ and κ- subtypes, which are inhibitory in nature and belong to the Gi/Go-coupled superfamily of receptors. It is highly conserved across vertebrates, including mammals and birds [1–3]. All three subtypes, μ, δ, and κ ORs have been reported to mediate analgesia [4, 5]. The opioid system is not only involved in physiological functions but is also involved in complex behaviors such as socialization [2, 6–8] and the motivation to perform different behaviors [9, 10].

Historically, opioids were first used for producing analgesia and opium [derived from poppies (*Papaver somnifera*)] was the first opioid drug used for pain relief from prehistoric times. Morphine and codeine, which are the active components of opium, mainly bind to μ-ORs. Over the years, many μ-OR agonists used for producing analgesia have been synthesized based on the structure of morphine [11, 12]. Since research on the opioid system began with the discovery of opium and its derivatives, much of the earlier literature on opioids has focused on μ-ORs and detailed studies of the δ-OR system are more recent. Besides μ-ORs, the δ-OR system also plays an important role in modulating various brain functions. Systemic administration of the δ-OR agonist SNC-80 has been found to impair learning and memory, produces anti-depressant-like effects in rats [13, 14] and relieve allodynia in a mouse model for migraine by binding to GABAergic neurons in the forebrain [15]. It is also known to mediate neuroprotection in hypoxic/ischemic stress via the BDNF-TrkB pathway by inhibiting excitatory neurotransmitter release, attenuation of disrupted neuronal transmission and stabilizing ionic homeostasis [16]. The three subtypes of ORs are also known to modulate movement since microinjections of their agonists or antagonists increase or decrease locomotor activity in rodents [17, 18]. Interestingly, intracerebroventricular injections of the δ-OR agonist DPDPE (D-Pen^2^, D-Pen^5^ enkephalin) led to a decrease in high frequency ultrasonic vocalizations (32–65 kHZ) in female rats, whereas infusions of naltrindole, which acts as a partial δ-OR agonist, led to a decrease in both high and low frequency (20–32 kHZ) vocalizations produced during aggressive interactions [19]. These findings demonstrate that the endogenous opioids and δ-ORs may play a role in vocalization. In studies on birds, cognitive processes such as learning, and memory are known to be modulated by the opioid system. For example, Patterson et al. demonstrated that injections of a δ-OR agonist [D-Pen2,L-Pen5] enkephalin (DPLPE)] into IMHV (Intermediate medial hyperstriatum ventrale), a part of the pallium in chicks, led to impairments on a taste aversion task [20].

Earlier studies have reported that the expression patterns of the three opioid receptor subtypes and their ligands differ widely in different regions of the brains of mammals, reptiles and birds [21–25]. Whereas levels of μ-OR mRNA are high in the striatum, VP (ventral pallidum), hippocampus, thalamic nuclei, hypothalamus, substantia nigra (SN), various nuclei of the brain stem, dorsal horn of the spinal cord, they are low in the prefrontal cortex in humans and rodents. In contrast, δ-OR mRNA is almost absent from SN, many brain stem nuclei, the dorsal horn of the spinal cord, diencephalon and is strongly expressed in the cortical layers, hippocampus and striatum. Additionally, levels of κ-OR mRNA are low to moderate in the cortex and striatum, high in some of the thalamic nuclei and hypothalamus and moderate to high in brainstem nuclei and SN [21, 22, 25].

We were interested in studying the expression of δ-ORs in zebra finches (*Taenopygia guttata*), a species of songbirds widely used as a model system to study the interactions between neural circuits and behavior [26, 27]. As seen in humans, songbirds learn their vocalizations during a sensitive period from a song model (generally, their fathers) and their songs are used as a mode for communication, courtship behavior, individual recognition and aggression [28–30]. The song control system in these birds comprises of the vocal motor pathway (VMP) and the anterior forebrain pathway (AFP) which is a basal ganglia loop and is involved in song learning and maintenance as well as context-dependent singing [23, 31, 32]. Besides AFP, the auditory pathway plays an important role in learning how to vocalize. During the sensory and sensorimotor phase, juvenile songbirds hear their tutors’ songs and an auditory template of this song is stored in the caudomedial nidopallium (NCM) [33]. The nidopallial nucleus, HVC receives auditory inputs from NIf, an auditory nucleus in the nidopallium, and projects to Area X of the AFP and RA of the VMP, linking auditory input to the vocal motor circuitry and the neural circuit essential for learning by providing a feedback loop [30, 34, 35]. Earlier studies have demonstrated that high levels of the opioid ligand enkephalin, which preferentially binds to δ-ORs (and to a lesser degree, to μ-ORs), are expressed in brain regions involved in singing, especially in the pallial (cortical) nuclei HVC and LMAN in these birds [36]. Other song control regions such as Area X [a nucleus of the basal ganglia consisting of a mixture of medium spiny neurons (MSNs) and larger pallidal neurons (Pd)] and the thalamic nucleus DLM are also immunoreactive for enkephalin. Additionally, areas such as GP (globus pallidus) and LSt (lateral striatum) which are part of the avian basal ganglia also demonstrate intense label for enkephalin [36, 37]. Interestingly, singing for half an hour induces the expression of proenkephalin, the precursor of enkephalin in HVC and Area X in zebra finches [38] and in the medial preoptic nucleus POM (medial preoptic nucleus) in male European starlings [39], suggesting that δ-OR activity as well as song-induced reward facilitates singing behavior, which may be crucial for song learning. Furthermore, song control areas of zebra finches express high levels of opioid receptor mRNA. Although both μ- and δ-ORs are present in most brain regions, the levels of δ-ORs are lower than those of μ-ORs [23]. An earlier study demonstrated that blocking ORs using the general opioid antagonist naloxone systemically leads to a decrease in directed and undirected songs and their spectro-temporal properties in adult male zebra finches [40]. Additionally, site-specific injections of naloxone in the anterior forebrain pathway of the song control system leads to changes in the number and acoustic features of female-directed song [10, 27]. Preliminary data from our lab has additionally demonstrated that blocking δ-ORs in adult birds leads to a decrease in female-directed singing and a slight decrease in song length but no major changes in the spectro-temporal properties of song [41].

Taken together, these results suggest that in songbirds, δ-ORs may play a role in song learning and the motivation to sing to females. However, the expression of δ-ORs has not been studied in the song control system of zebra finches. We found that there was a high degree of sequence similarity between the different opioid receptor subtypes in zebra finches. The δ-OR protein (XP_012426093.3) has a ~70% sequence identity with the μ-OR protein (XP_030123902.1) and a ~63% sequence identity with the κ-OR protein (XP_012427437.3) in these birds [42–44]. Due to this high sequence homology, most commercially available antibodies do not specifically detect the δ-OR protein. Therefore, we decided to study its expression in the zebra finch brain by performing fluorescence in-situ hybridization (FISH) using a specific riboprobe designed to detect δ-OR mRNA.

## Methods

A total of 6 adult male zebra finches were used for these experiments. The experimental protocols were approved by the Institutional Animal Ethics Committee, National Brain Research Centre, Manesar according to the guidelines laid down by the Committee for the Purpose of Control and Supervision of Experiments on Animals (CPCSEA), India, which are compliant with international standards on animal welfare to minimize pain and discomfort to the birds. Birds were housed in an indoor aviary at a 12 h light and dark cycle with a temperature ranging from 25ºC-29ºC throughout the year and were provided ad libitum access to food and water.

### Perfusion and cryosectioning

Zebra finches (n = 6) were deeply anaesthetized with ketamine (30mg/kg) and xylazine (2 mg/kg) and perfused transcardially with 0.01M PBS (phosphate buffered saline in DEPC treated Milli Q water) followed by 4% PFA (paraformaldehyde). Brains were post-fixed in 4% PFA for 24–36 hours at 4º C and kept in 30% sucrose until they were completely saturated with sucrose for cryoprotection. Ten-micron thick coronal serial sections of the brains were cut using a cryostat (Leica) and mounted on positively charged VWR superfrost Plus slides (Cat. No. 4811–703). One series was stained with Nissl and the others were used for fluorescence in-situ hybridization (FISH).

### Fluorescence in-situ hybridization (FISH)

Specific sequences corresponding to the zebra finch δ-ORs were used from the NCBI Genbank. Forward and reverse primers designed against the predicted sequence for the zebra finch δ-OR (Accession number: XM_012570639.3) are TTCAACCTGGCTCTGGCTGATG and GTCAATAGAGAGCACAACCTTGC. Amplicons specific to δ-ORs were cloned in the pCR II vector [Invitrogen; PCR cycle: (1) Denaturation: 95 ºC for 15min; (2) Annealing (40 cycles): i) 95 ºC for 30 sec, (ii) 62 ºC (melting temperature) for 30 sec, (iii) 72 ºC for 40 sec; (3) Extension: 72 ºC for 10min]. The vector was linearized with restriction enzymes BamH1 and EcoRV individually and transcribed with T7 and SP6 polymerases to generate DIG-labeled antisense and sense riboprobes.

Sections were rinsed in DEPC-treated Milli Q water and fixed in 4% PFA for 15 min after which they were permeabilized in 0.2 N HCl for 10 min and washed with GB1 (Tris-saline) buffer for 5 min. After acetylation for 10 min and permeabilization with 15μg/ml proteinase K for 10 min at 37°C, sections were washed twice in a solution containing 0.01M PBS and 100mM glycine for 3 min each, followed by three washes with chilled GB1 for 5 min each. Serial dehydration was performed by treating sections sequentially with ascending grades of ethanol and then with chloroform. This was followed by incubation in a prehybridization buffer containing sense or antisense riboprobes (1.5ng/μl) at 75°C for 10 min and then at 45°C in a humid chamber for 16 to 20 hr for hybridization. Slides were washed with 2X, 0.2X, 0.02X and 0.002X SSC (saline sodium citrate) containing 0.05% Tween- 20 at 40°- 45° C for 10 min each, followed by washing in 0.002X SSC + 0.05% Tween-20 for 5 min. Blocking was performed by incubating sections in 0.5% blocking reagent (Cat. No. FP1020, TSA kit Perkin Elmer) for 30 min followed by 2 hr incubation in anti-DIG peroxidase (1:250, Cat. No. 11207733910, Roche). This was followed by two rinses with GB1, after which sections were treated with biotinyl tyramide (Cat. No. SAT700001EA, Perkin Elmer) for 10 min. Sections were again washed with GB1 and treated with Streptavidin-Fluorescein for 30 min [23]. Finally, sections were washed and coverslipped with ProLong^™^ Gold Antifade Mountant with DAPI (P36935, ThermoFisher Scientific).

### Fluorescence microscopy

We imaged entire sections stained for δ-OR mRNA at a magnification of 4X using Neurolucida software (version 2020.1.3; MBF Bioscience, USA) linked to a fluorescence microscope (Olympus BX53). Song control and auditory areas including LMAN, Area X, DLM, HVC, RA, NIf, Ov (nucleus ovoidalis) and MLd (Schematic S1 Fig) were imaged at a magnification of 10X using an Apotome microscope (Carl Zeiss^®^, AxioImager.z1). For quantitative measurements, Z-stacks of 5 neurons from each area were captured at a magnification of 63X from each bird. Single sections in each Z-stack were 0.24μm thick. The exposure time was decided by the staining intensity of hippocampal cells (which acted as a control for normalization) and was kept constant for the areas within the same section.

#### Intensity measurements

All measurements were performed using ImageJ software (version 1.48). An ROI was drawn around individual cells in each frame (5 frames / cell) of the Z-stack and their intensity was measured. Background pixels were subtracted from the intensity measurements. A CTCF (corrected total cell fluorescence) value was calculated for each frame (https://theolb.readthedocs.io/en/latest/imaging/measuring-cell-fluorescence-using-imagej.html) by using the formula:

$$\text{CTCF}\;=\;\text{Integrated}\;\text{Density}\;-\;\left(\text{Area}\;\text{of}\;\text{selected}\;\text{cell}\right)\;\times \;\left(\text{Mean}\;\text{fluorescence}\;\text{of}\;\text{background}\;\text{readings}\right)$$

The mean of the CTCF values from all frames were normalized by CTCF values from the hippocampus (Hi2 area) in the same section. Intensity measurements from hippocampal cells were used for normalization since it extends along the rostro-caudal extent of the zebra finch brain and was present in all sections used for our analysis. Furthermore, it is a non-song control region and is well-suited to act as a control.

#### Measurements of cell size in Area X

Small (S) and large (L) striatal neurons in Area X were identified based on the diameters of their perikarya in Z-stacks used for intensity measurements, as described previously [45]. The longest and shortest axes of the 25 small and 25 large neurons (from 5 birds, used for intensity measurements) were measured using ImageJ (version 1.48) and were averaged.

### Statistics

We used SigmaPlot 14.0 (Systat Software, San Jose, CA) for statistical analyses. The Shapiro-Wilk normality test and the Brown-Forsythe equal variance test were performed before proceeding to hypothesis testing. Parametric tests were performed if the data followed a normal distribution and demonstrated equal variance, failing which non-parametric tests were performed to test for significant differences. A One-Way Repeated Measures ANOVA (RM-ANOVA) or Friedman ANOVA on ranks (in case the data violated the test assumptions) was performed on the normalized collated intensity data for individual neurons of different brain regions from all experimental birds. We compared song control regions, auditory areas, and also performed an overall comparison of the pallial areas versus song control areas or auditory areas using One-Way RM-ANOVA. A Kruskal-Wallis ANOVA on ranks was performed only in the case of multiple comparisons for auditory areas since data from NIf was only available in n = 4 birds. We also performed paired t-tests, or the Wilcoxon signed rank test (two-tailed) for comparing intensity measurements from two areas. Wherever there were unequal numbers of data points in the two categories, a Welch’s t-test or a non-parametric Mann-Whitney test was performed. We used α = 0.05 as a threshold P-value to report significance.

## Results

### Riboprobe specificity for δ-ORs in zebra finches

The primers used in our study generated a single PCR product of 120 bp (TTCAACCTGGCTCTGGCTGATGCAGTGGCCACCAGCACACTGCCCTTCCAGAGCACCAAGTACCTCATGGAGACCTGGCCCTTTGGGGAGCTGCTCTGCAAGGTTGTGCTCTCTATTGAC) which was specific for the OPRD1 cDNA (XM_012570639.3) predicted sequence in the NCBI database [42]. The designed sequence demonstrated 100% sequence identity, an E value of 3e-62 and a total score of 222 when aligned with the predicted sequence. The predicted OPRD1 cDNA sequence has a sequence identity of 73–74% with the two predicted OPRM1 cDNAs (μ-OR RNAs). However, we did not find any significant sequence similarity between the designed probe with the predicted OPRM1 cDNA sequence (XM_030268042.2, XM_030268041.2). Furthermore, staining with the sense probe that we have designed as a control produced negligible label in sections stained using fluorescent in-situ hybridization (S2A and S2B Fig).

### Expression of δ-OR mRNA in the zebra finch song control system

**Fig 1:**
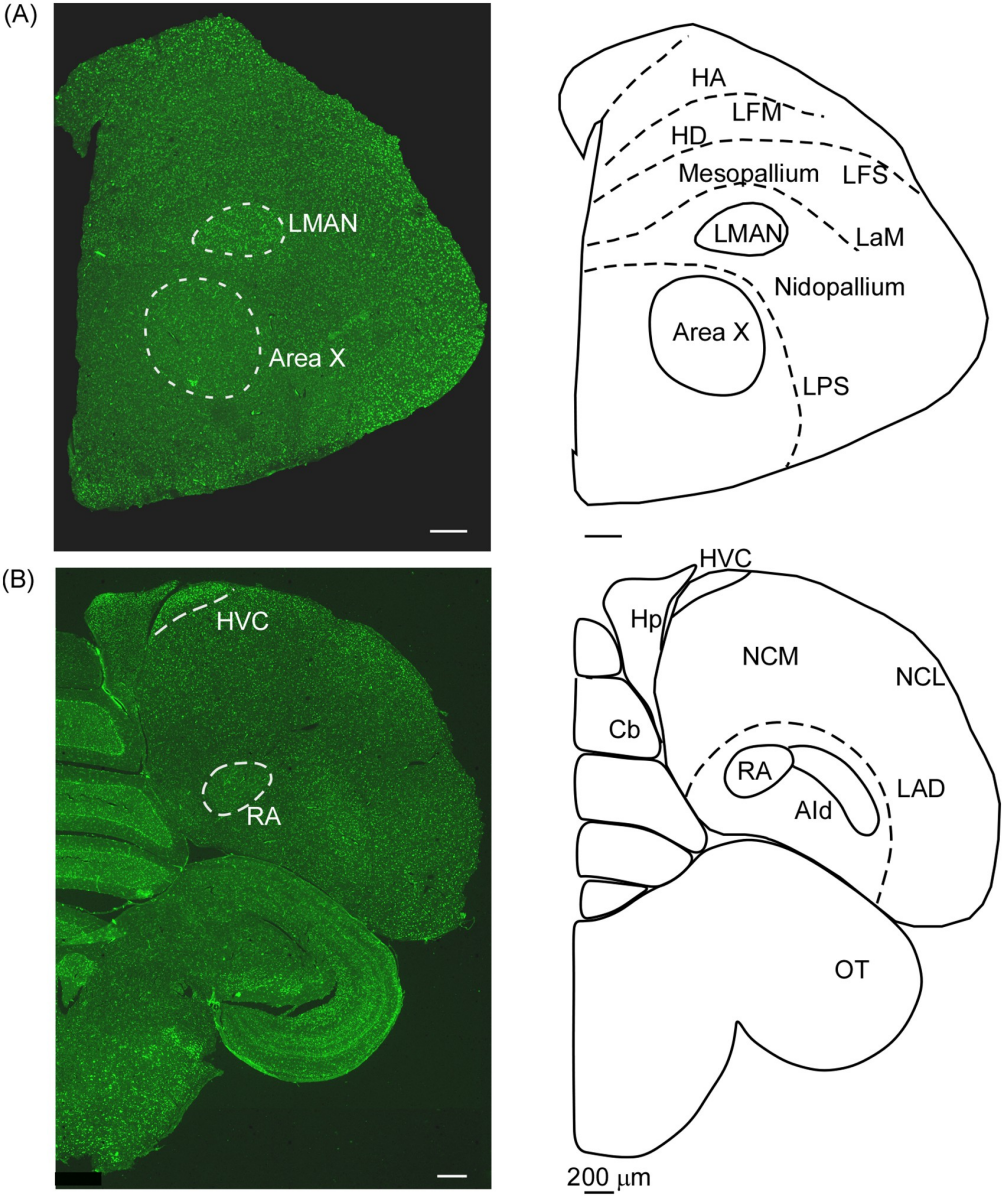
Coronal brain sections at the level of song control areas. (A) At the level of the anterior forebrain, song control areas LMAN and Area X are clearly visible at low magnification. Whereas LMAN is intensely labelled for δ-OR mRNA, Area X and the surrounding MSt have lower levels of staining compared to the surrounding nidopallium. (B) At the level of the posterior forebrain, HVC is clearly visible as a result of high levels of δ-OR mRNA expression. Whereas the boundaries of the vocal motor nucleus RA can also be clearly demarcated, the staining intensity was lower in this area. Scale bar, 200 μm. AId, Dorsal intermediate arcopallium; Cb, Cerebellum; HA, Hyperpallium apicale; HD, Hyperpallium dorsale; Hp, Hippocampus; LAD, Lamina arcopallialis dorsalis; LaM, Lamina mesopallialis; LFM, Lamina frontalis suprema; LFS, Lamina frontalis superior; LMAN, Lateral magnocellular nucleus of the anterior nidopallium; LPS, Lamina pallio-subpallialis; NCL, Caudolateral nidopallium; NCM, Caudomedial nidopallum; OT, Optic tectum; RA, Robust nucleus of arcopallium.

**Fig 2:**
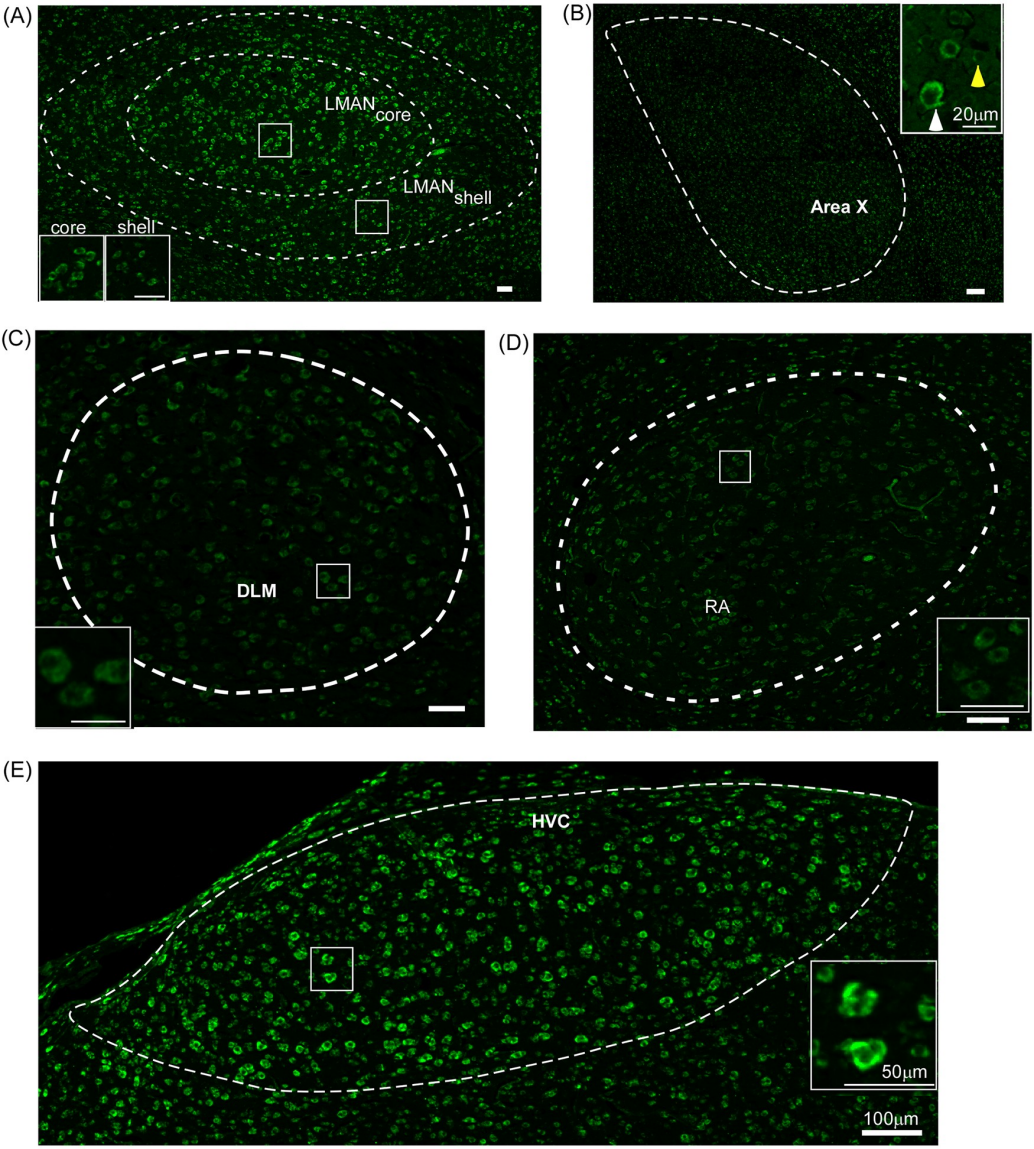
The expression of δ-OR mRNA in song control areas. (A) In the anterior forebrain pathway, magnocellular neurons in the central (core) region and parvicellular (shell) neurons of the nidopallial song control nucleus LMAN express high levels of the δ-OR mRNA (insets show staining in individual neurons in each area). (B) Area X demonstrated lower levels of staining compared to the overlying nidopallium. The level of δ-OR mRNA expression in the Area X(L) neuron (*white arrowhead*) was significantly higher than that in a smaller neuron [Area X(S); *yellow arrowhead*] in this region. (C) DLM, a thalamic nucleus which is also a part of the AFP and projects to LMAN, is weakly stained for δ-ORs. (D) and (E) Song control nuclei (RA and HVC) of the vocal motor pathway also demonstrate δ-OR mRNA expression. Whereas neurons in HVC are strongly labelled, those in RA express lower levels of staining. Scale bars, 100 μm for low magnification images and 50 μm for insets. DLM, Dorsolateral nucleus of the medial thalamus; LMAN, Lateral magnocellular nucleus of the anterior nidopallium; RA, Robust nucleus of arcopallium.

**Fig 3:**
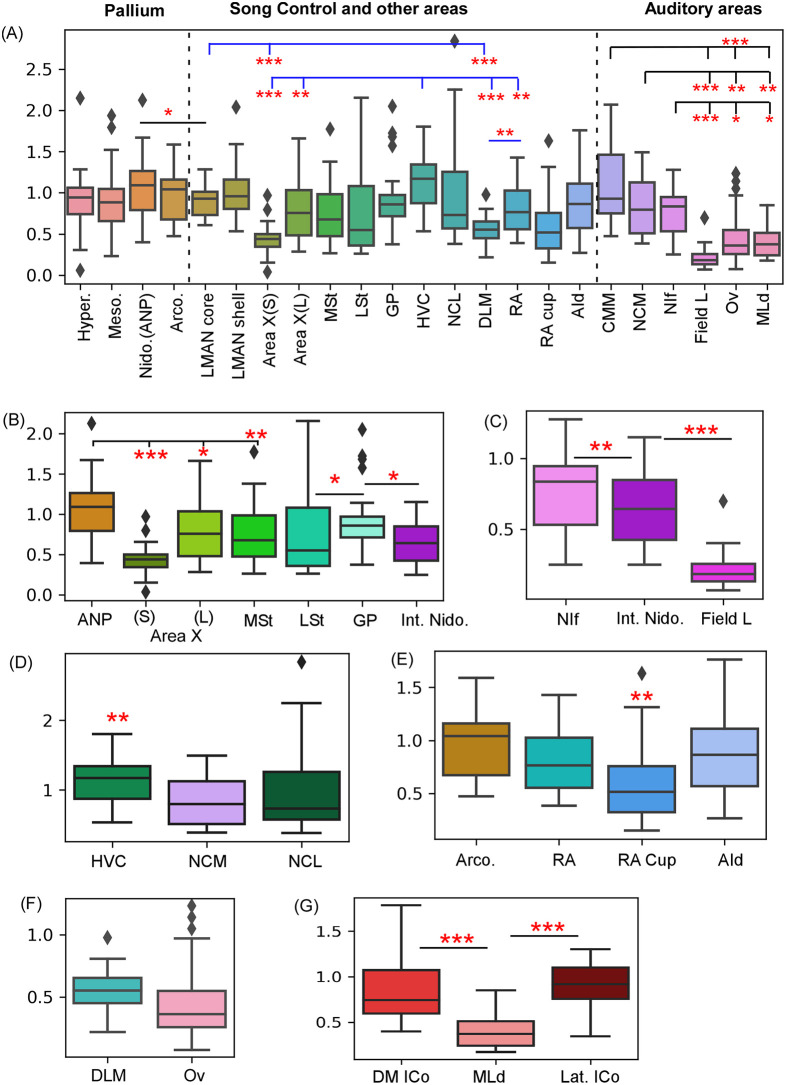
Quantification of δ-OR mRNA in the zebra finch brain. (A) A comparison of δ-OR mRNA levels across various regions in the brain. In the boxplots used here, boxes represent the interquartile range, whiskers represent the maximum and minimum values in the data and the outliers are represented by data points above and below the whiskers. Amongst the song control areas, neurons were most intensely labeled in HVC and LMAN whereas those in DLM, Area X and RA have lower levels of label for δ-OR mRNA (statistical comparison indicated by blue line). Auditory cortical areas (separated by a dashed line from other brain regions) also demonstrate comparably high levels of label at the level of CMM, NCM and NIf. Levels of label in these regions were significantly higher than those in the thalamic nucleus Ov and MLd (a midbrain nucleus), whereas the lowest levels of δ-OR mRNA were present in Field L. (B) In the anterior forebrain, areas of the avian basal ganglia, that is, MSt and Area X demonstrate weak labeling and the surrounding nidopallium is comparatively strongly labeled. At more caudal levels, neurons in GP were intensely labelled compared with those in LSt and the intermediate nidopallium (Int. Nido.). (C) At an intermediate level along the rostro-caudal extent of the brain, the auditory nucleus NIf demonstrates higher levels of label for δ-OR mRNA than the surrounding Int. Nido. whereas Field L demonstrates the least. (D) The song control nucleus HVC can be clearly demarcated from the surrounding nidopallium. (E) The lowest levels of label for δ-OR mRNA are present in the RA cup region compared to the surrounding arcopallium and the song control areas RA and AId. (F) Comparably low levels of δ-OR mRNA were present in the thalamic nuclei DLM and Ov. (G) Cells in the midbrain auditory nucleus MLd demonstrated lower staining intensity for δ-ORs than the surrounding subdivisions of ICo. ***, P<0.001; **, P<0.01; *, P<0.05. ANP, Anterior nidopallium; AId, dorsal intermediate arcopallium; CMM, caudomedial mesopallium; DLM, Dorsolateral nucleus of the medial thalamus; DM ICo, dorsomedial part of nucleus intercollicularis; LMAN, Lateral magnocellular nucleus of the anterior nidopallium; GP, Globus pallidus; LSt, Lateral striatum; MLd, Nucleus mesencephalicus lateralis, pars dorsalis; MSt, Medial Striatum; NCL, Caudolateral nidopallium; NCM, Caudomedial nidopallium; NIf, Nucleus interfaciallis nidopalli; Ov, Nucleus ovoidalis; RA, Robust nucleus of arcopallium.

The δ-OR mRNA expression was found in the cytosol in neurons throughout the zebra finch brain. There were significant differences in the levels of δ-OR expression across various song control regions (Figs 1 and 2). We found that LMAN [mean difference: -0.167 (0.358)] and HVC [mean difference: 0.287 (0.358)] were clearly demarcated from the surrounding anterior and caudal nidopallium, respectively (t = -2.339, P = 0.028 for LMAN, paired t-test; P = 0.001; Friedman RM ANOVA on Ranks for HVC, P = 0.005 (NCL) and P = 0.003 (NCM), Tukey post hoc test; Figs 2A and 2E and 3A and 3D; intensity measurement data provided as S1 File). Whereas both magnocellular neurons of LMAN_core_ and parvicellular neurons in LMAN_shell_ expressed δ-OR mRNA, label was more intense in LMAN shell neurons compared to that in LMAN_core_ (ns; P = 0.0842, paired t-test; Figs 2A and 3A).

We performed intensity measurements of the two cell types in Area X based on their perikaryal diameter in optical sections and found that the diameter of the larger somata [Area X(L); diameter data provided in S1 File] was 17.57 ± 2.81 μm and that of the smaller neurons [Area X(S)] was 7.02 ± 0.98 μm. The size of the Area X(S) neurons was similar to the small GAD-positive (GAD; glutamic acid decarboxylase) neurons reported by Carrillo and Doupe (2004) and DARPP-32 and Substance P-labelled striatal spiny neurons (MSNs) reported by Reiner et al. (2004), using immunohistochemistry. However, based on their size, the smaller δ-OR-positive neurons could be MSNs or striatal interneurons which were either exclusively parvalbumin-positive or somatostatin-positive [45]. We found that the diameter of Area X(L) δ-OR-positive neurons in our study was larger than that previously reported (~13 μm) for LANT6-positive pallidal neurons in Area X or ChAT (choline acetyltransferase) striatal interneurons [45]. In the Carillo and Doupe (2004) study, larger GAD-positive neurons were 14–20 μm in diameter and ChAT-positive interneurons were ~15 μm in diameter. It is therefore possible that the larger δ-OR-labeled neurons in our study are pallidal neurons or alternatively, ChAT-labeled interneurons.

Amongst the vocal control regions, the intensity of label for δ-ORs was the highest in HVC and it was significantly higher compared to both Area X(S) and Area X(L), RA and DLM [RM-ANOVA, F_(5,119)_ = 20.773; P<0.001; post hoc Tukey test, P<0.001 (Area X, DLM), P = 0.002 for RA; **[**Fig 3A]. The staining intensity was second highest in LMAN, with neurons in LMAN_core_ demonstrating more intense label than in Area X(S) neurons and neurons within DLM (Tukey post hoc test, P<0.001). Furthermore, label was higher in HVC compared with that in LMAN_core_ (Tukey post hoc test, P = 0.034). In Area X, larger neurons (L) were more intensely labelled than smaller (S) ones (Tukey post hoc test, P < 0.001). Amongst the song control areas, the lowest levels of δ-OR mRNA were detected in DLM, which were lower than those in RA (Tukey post hoc test, P = 0.005). Our results therefore demonstrate that the order of δ-OR mRNA expression in song control area is HVC > LMAN > Area X(L), RA > DLM > Area X(S).

### Expression of δ-ORs in subdivisions of the basal ganglia

**Fig 4:**
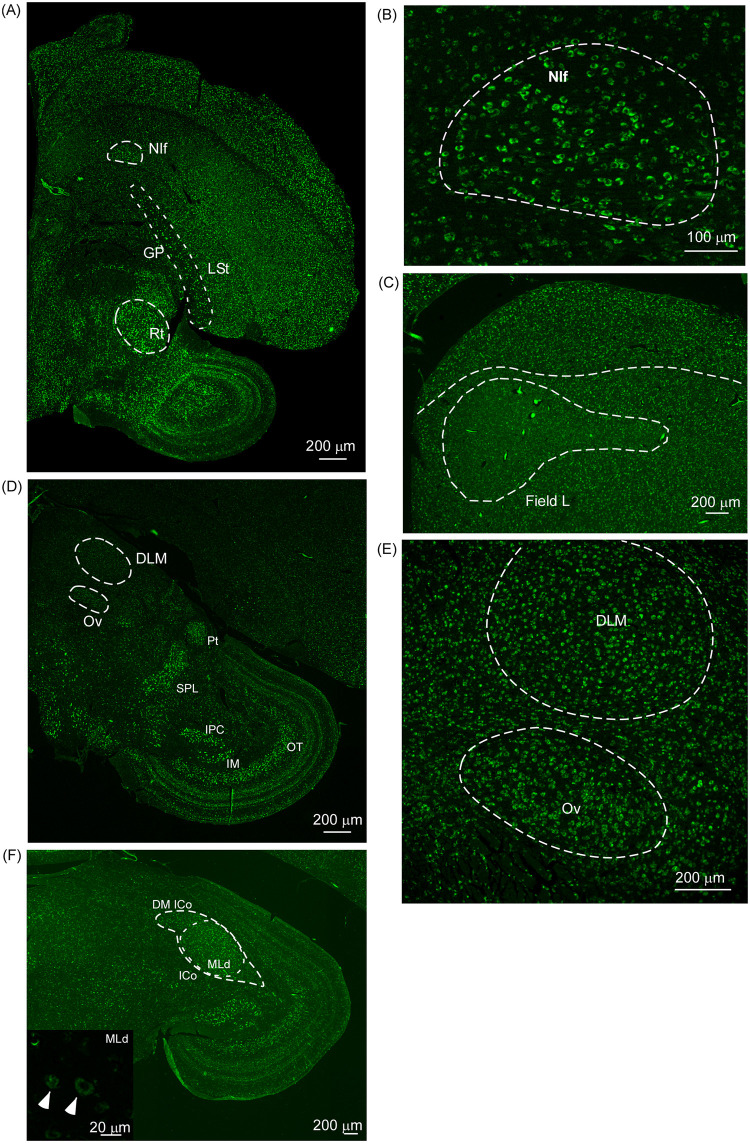
The expression of δ-OR mRNA at an intermediate plane along the rostro-caudal extent of the brain. (A) At low magnification, parts of the basal ganglia (the LSt and GP) and the auditory area NIf are clearly visible based on higher levels of δ-OR mRNA expression. (B) The auditory nucleus NIf can be clearly demarcated from the surrounding intermediate nidopallium (INP). (C) Field L is clearly visible as a highly myelinated area but with low levels of δ-OR mRNA. (D) The thalamic nuclei DLM (a part of the song control system) and Ov (a part of the auditory system) are clearly visible based on the presence of low levels of δ-OR mRNA in these regions. (E) A high magnification image of DLM and Ov demonstrates that the boundaries of both thalamic nuclei are clearly visible and can be easily delineated from the surrounding thalamus. (F) The auditory nucleus (MLd) can be easily delineated from surrounding midbrain based on high levels of myelination. However, individual neurons in this region are weakly labelled, as can be seen in the inset at higher magnification. Scale bar for A, C, D, E and F, 200μm, scale bar for B, 100μm and scale bar for the inset in F, 20μm. DLM, Dorsolateral nucleus of the medial thalamus; DM ICo, dorsomedial part of nucleus intercollicularis; GP, Globus pallidus; IM, Nucleus isthmi, pars magnocellularis; IPc, Nucleus isthmus, pars pavocellularis; LSt, Lateral striatum; MLd, Nucleus mesencephalicus lateralis, pars dorsalis; NIf, Nucleus interfaciallis nidopallii; Ov, Nucleus ovoidalis; OT, Optic tectum; Pt, Nucleus pretectalis; Rt, Nucleus rotundus; SPL, Nucleus spiriform lateralis.

The medial striatum (MSt), located in the anterior forebrain ventral to LMAN, could be clearly demarcated from the anterior nidopallium (ANP) based on low levels of δ-OR mRNA expression in this region (RM-ANOVA, F_(3,71)_ = 20.298, P<0.001, Fig 3B and S3A Fig). For our intensity measurements, we sampled a mixed population of neurons in MSt, which included all subtypes (based on size and morphology) besides pallidal neurons [45, 46]. Furthermore, levels of δ-OR expression were also low in Area X compared with those in the overlying anterior nidopallium (Tukey post-hoc test, 0.030 for Area X(L) and P<0.001 for Area X(S); Fig 3B). Neurons in MSt demonstrated more intense label in comparison with Area X(S) neurons (Tukey post hoc test, P = 0.002). Smaller neurons, which were most abundant in Area X were weakly labelled by δ-OR mRNA compared with the larger neurons present in this nucleus (as shown at higher magnification; Fig 2B). Other parts of the avian basal ganglia, namely, the LSt which is located at intermediate levels along the rostro-caudal extent of the zebra finch brain also demonstrated low staining intensity, whereas neurons in the GP were more strongly labelled (Signed rank test, P = 0.034; Figs 3B and 4A and S4A and S4B Fig). The intensity of label in GP was even higher than the surrounding nidopallium (Int. Nido.; Mann-Whitney Rank Sum Test, U = 142.000, P = 0.014).

### Expression of δ-OR mRNA in auditory areas

We observed that auditory areas, caudomedial mesopallium (CMM), caudomedial nidopallium (NCM) and nucleus Interfacialis nidopallii (NIf) were intensely labelled for δ-OR mRNA whereas Field L, nucleus ovoidalis (Ov) and MLd were weakly labelled (Figs 3 and 4 and S4D–4F Fig). Whereas the boundaries of the thalamic nucleus (Ov) and midbrain nucleus MLd could be easily delineated from the surrounding regions, individual neurons in these nuclei were weakly labelled (Fig 4E and 4F). The mesopallial area CMM was the most intensely labelled for δ-OR mRNA amongst different components of the auditory pathways (Kruskal-Wallis ANOVA on ranks; H = 81.199, P = <0.001; Dunn’s post hoc test, P <0.001 for Field, Ov and MLd; Fig 3A). The caudomedial nidopallium was also labelled intensely for δ-OR mRNA compared with Field L, Ov and MLd (Dunn’s post hoc test; P<0.001 for Field L, P = 0.004 for Ov and P = 0.001 for MLd). Label in NIf was significantly higher compared to the surrounding intermediate nidopallium (Int. Nido.; t = 3.234, P = 0.004, paired t- test; Figs 3C and 4A and 4B) and also significantly higher than that in Field L, Ov and MLd (Dunn’s post hoc test; P<0.001 for Field L, P = 0.037 for Ov and P = 0.014 for MLd; Fig 3A). The primary auditory cortex, Field L, was clearly visible from the surrounding nidopallium based on lower levels of label and higher levels of myelination (t = 6.408, P = 0.000000740, Welch’s t-test; Fig 4C). Furthermore, we were unable to delineate the auditory nucleus, nucleus Avalanche (Av) from the surrounding caudal mesopallium in our stained sections. The thalamic nucleus Uva (which provides input to HVC) was also unidentifiable in our sections, suggesting that neurons in these regions do not express significant levels of δ-ORs. We were therefore unable to quantify levels of δ-ORs in these areas. Based on these findings, the order for δ-OR mRNA expression across components of the auditory pathways is CMM > NCM > NIf > Ov, MLd > Field L.

### Comparison of δ-OR mRNAs in MLd and ICo

The MLd is surrounded by the intercollicular nucleus ICo, which when stimulated, leads to the production of vocalizations in a number of avian species [reviewed in [47]]. We found that both dorsomedial and lateral divisions of ICo expressed significantly higher levels of δ-OR mRNA in zebra finches, compared to that in MLd (Kruskal-Wallis ANOVA on ranks; H = 35.899; P <0.001; Dunn’s post hoc test; P <0.001 for both dorsomedial and lateral ICo versus MLd).

### Expression of δ-OR mRNA in other brain areas

Whereas all pallial areas including the hyperpallium, mesopallium, nidopallium, NCL, NCM and arcopallium expressed δ-OR mRNA, levels of expression were not significantly different (RM ANOVA, F_(5, 115)_ = 0.799, P = 0.552; S3 and S4C, S4D and S4H Figs). At the level of the anterior forebrain, this expression was slightly higher in the nidopallium compared with that in the hyperpallium and mesopallium (ns; Fig 3A and S3B–3D Fig). In the caudal telencephalon, both NCM and NCL regions were composed of δ-OR mRNA positive neurons (Fig 3). We also found that the different parts of hippocampal complex, that is, the dentate gyrus, hippocampal area 2 (Hi2; shown in S3E Fig), the parahippocampal area (APH) and the dorsolateral corticoid area (CDL; not shown) were positive for δ-OR mRNA, but since the hippocampus was taken as a control for normalization, we did not quantify the level of δ-OR mRNA expression in these regions.

In the arcopallium, the RA cup and AId, a region located at the caudal-most part of the brain and involved in singing, auditory and visual motor behavior [48–50] was also positive for δ-OR mRNA. Amongst arcopallial areas, the RA cup demonstrated lower levels of label compared to that in the arcopallium, RA and AId (RM ANOVA, F_(3, 62)_ = 7.733, P ≤0.001, Tukey post-hoc test, RA cup versus arcopallium, P<0.001; RA cup versus AId, P = 0.001 and RA cup versus RA, P = 0.003 with RA; Fig 3E and S3F and S4G and S4H Figs). The three layers of the cerebellum displayed variable staining patterns for δ-ORs. All neurons in the Purkinje cell layer were strongly labelled, whereas cells in the molecular and granular layer were intensely labelled but were distributed sparsely (S2C Fig).

## Discussion

We have used in situ hybridization to demonstrate that δ-OR mRNA is widely expressed in the zebra finch brain. The riboprobe that we designed was specifically targeted to δ-OR mRNA and did not overlap with sequences of other major OR subtypes (μ- or κ-), unlike that used in an earlier study [23] which detected both μ- and δ-ORs. In the present study, we have focused on components of the song control and auditory systems and have provided comparisons between components of the pallium and basal ganglia in terms of the levels of δ-OR expression in these areas. In the following sections, we have discussed our findings vis-à-vis the localization of the ligand for δ-ORs (met-enkephalin) in adult male zebra finches [36] and have also provided comparisons between δ-OR expression in songbird [juncos; [51]] and non-songbird species [pigeon, [52] and chick [53]], wherever possible. A caveat is that these findings are not directly comparable to ours, since we have used in situ hybridization, whereas met-enkephalin was detected using immunohistochemistry and studies on the expression of δ-ORs in juncos, pigeons and chicks had employed autoradiography.

### Expression of δ-ORs in pallium, basal ganglia and thalamus

#### Pallium

**Fig 5:**
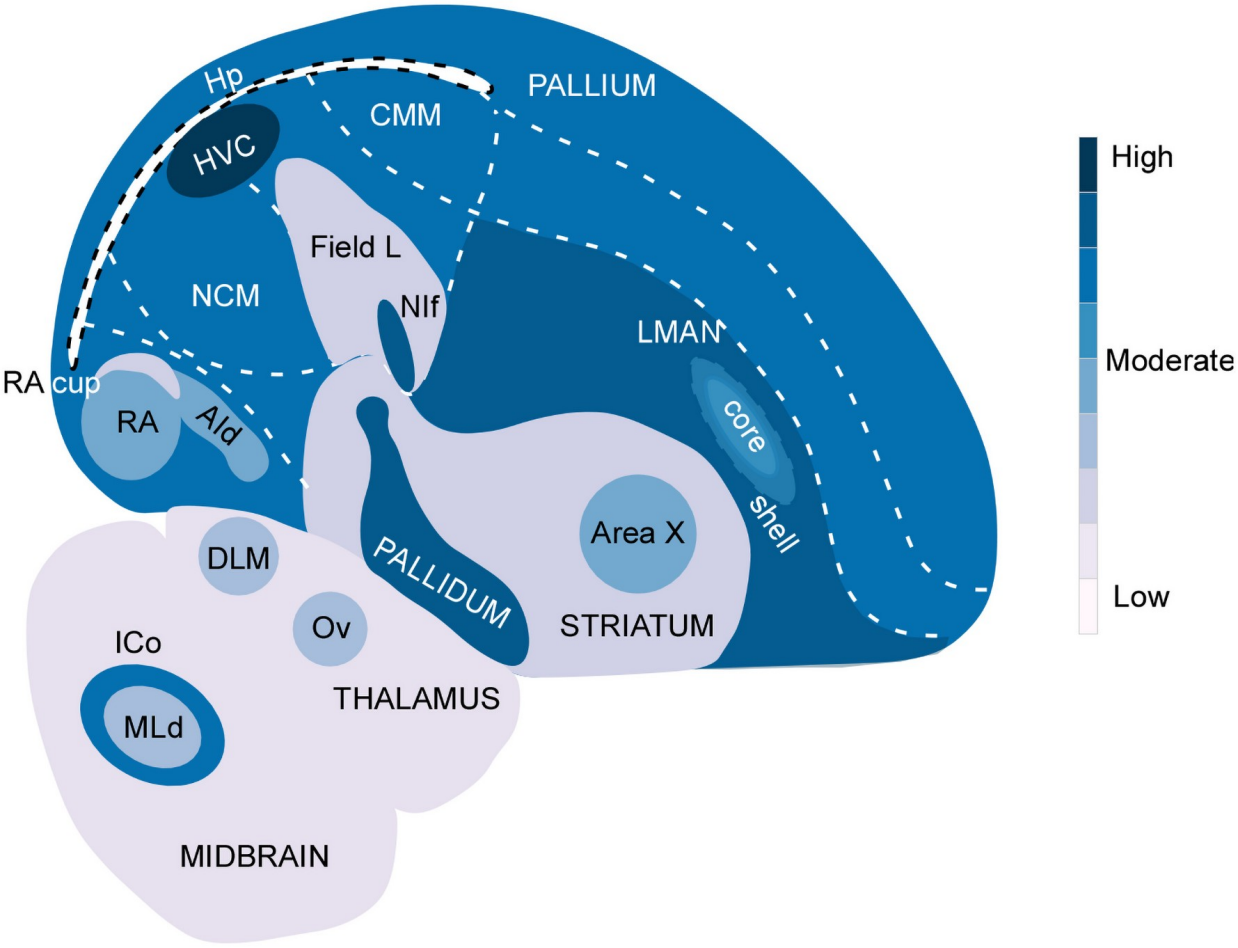
Schematic demonstrating δ-OR mRNA expression in the zebra finch brain. A summary of the pattern of δ-OR mRNA expression in various regions observed in our study. Dark blue indicates areas with the most prominent label, whereas light blue indicates regions with low staining intensity.

We found that δ-ORs were expressed in all divisions of the zebra finch pallium, including the hyperpallium, mesopallium, nidopallium and arcopallium (Fig 5). We also found that levels of δ-ORs in pallial areas were significantly higher than those in components of the basal ganglia underlying each area. That is, levels of δ-OR mRNA were higher in the anterior nidopallium than in the song control region Area X (except for large neurons, see below) and the surrounding MSt and in the intermediate nidopallium compared to that in LSt. These findings are similar to those in mammals wherein δ-OR mRNA [21] and *OPRD1* gene transcripts (humans, [54]) are significantly higher in the neocortex, parts of the allocortex including the entorhinal and endopiriform cortices, cortical amygdala and parts of the hippocampal complex than in the basal ganglia, except in cholinergic neurons in the mammalian striatum [55–57]. The presence of high levels of δ-ORs in these regions suggested that they played a role in modulating cognitive functions associated with the cortex, hippocampus, amygdala and basal ganglia. For example, Le Merrer et al. [58] has demonstrated that learning which is dependent on the hippocampus (tested using novel object recognition and dual-solution cross maze tasks) is disrupted in δ-OR knock-out mice or mice treated with the δ-OR antagonist naltrindole. Both sets of mice were also tested on tasks which were dependent on the striatum (a single-solution response cross-maze task and a motor skill-learning task). Their results demonstrated that striatal-based learning was facilitated in experimental animals versus controls, suggesting that δ-ORs inhibit striatal function and maintain a balance between hippocampal and striatal-based memory. Interestingly, we also found high levels of δ-OR expression in the hippocampal complex in zebra finches, suggesting that δ-ORs in birds may modulate hippocampal versus striatal-based memory. It is also possible that δ-ORs in the avian hippocampus modulate spatial learning [58–60].

#### Basal ganglia and cerebellum

We have discussed δ-OR expression in the basal ganglia and cerebellum in the same section, since both are involved in motor coordination and locomotion. Details of δ-OR expression in Area X at the cellular level are discussed in the section on the song control system.

We found that parts of the avian basal ganglia, namely the MSt and LSt, expressed low levels of δ-OR mRNA. The boundary of the song control nucleus Area X was very faintly distinguishable from the surrounding MSt. Additionally, large neurons in Area X, which could be either Area X pallidal projecting neurons or ChAT-positive interneurons based on their diameter, and GP were intensely labelled with δ-OR mRNA. Whereas there was uniform δ-OR expression throughout the songbird striatal areas, δ-ORs were abundantly expressed by inhibitory striatal projecting neurons present within ‘patches’ or striosomes embedded within a matrix in the rodent striatum [61]. Besides the striosomes, moderate levels of δ-ORs are expressed throughout the rat striatum [56, 62] and are highly enriched in striatal cholinergic interneurons, projection neurons rich in acetylcholine in the medial septum, and diagonal band of Broca [54–57, 63]. The presence of δ-ORs in the striatum suggests that they play a role in the coordination and production of movements and also in motor learning, besides reward and motivation. For example, Bertran-Gonzalez et al. [57, 64] demonstrated an increase in δ-ORs expressed by cholinergic neurons in the shell of the nucleus Accumbens in rodents following Pavlovian training in rats.

Besides δ-OR expression in the basal ganglia, we observed intensely stained Purkinje cells, sparse label in the molecular layer and negligible label in the granular layer of the cerebellum. This expression pattern was also observed in the cerebellum in rats and suggests a role for δ-ORs in modulating movement [65]. These findings suggest that δ-ORs can affect different components of the motor circuits across different species.

#### Thalamus

Levels of staining for the δ-OR mRNA were low in nuclei of the dorsal thalamic zone, including the song control nucleus DLM (see below), Uva and Ov in zebra finches (present study). Although not analyzed in our study, we found that nucleus Rotundus (Rt) was moderately labeled and could be clearly discerned from the surrounding areas based on δ-OR expression. Our results are similar to those in chicks, wherein Rt was the only thalamic nucleus to be heavily labeled for δ-ORs whereas the rest of the thalamus was unlabeled [33, 53, 66, 67], and also to the negligible expression of δ-ORs in the human thalamus [21]. In contrast to these findings, Reiner et al. [52] had shown that the dorsal thalamic zone expresses high levels of δ-ORs in pigeons, suggesting that δ-ORs may function differently in modulating the thalamus across various avian species.

### Expression of δ-ORs in the song control system of zebra finches

#### Vocal motor pathway

The vocal motor pathway controls movements of the respiratory and syringeal muscles, required for coordinating breathing and singing. It consists of projections from HVC to RA and from RA to the tracheosyringeal part of the hypoglossal nucleus which controls syringeal musculature [67–69]. Our results demonstrate that amongst the song control nuclei, the expression of δ-ORs was the highest in HVC and LMAN (discussed with other components of the Anterior Forebrain Pathway, see below), and could be easily discerned from the surrounding nidopallium. These findings are also similar to those published earlier, which had used a riboprobe to detect both μ- and δ-ORs in adult male zebra finches [23]. Bottjer and Alexander [36] have shown that HVC possesses a number of enkephalin-positive fibers, but few enkephalin-labeled somata. Since HVC controls temporal aspects of singing and the sequence in which individual notes are produced [70, 71], our findings suggest that temporal features of singing may be modulated by the δ-OR system. Since HVC also projects to Area X, δ-ORs may affect the AFP via the HVC → Area X pathway. Both the auditory nucleus NIf [34] and the nucleus Avalanche [35] which provide input to HVC also express high levels of δ-ORs, although the boundaries of nucleus Avalanche (Av) cannot be delineated from the surrounding CMM. Amongst the other inputs to HVC, the thalamic nucleus Uva does not appear to possess δ-ORs or enkephalin [36] and the auditory cortical area Field L is also not highly labeled for either δ-ORs or enkephalin-positive somata and fibers [36]. Although Bottjer and Alexander, (1995) have not commented on the density of enkephalin-labelled somata in either NIf or Av, they demonstrated a high density of enkephalin-labeled fibers and somata in MMAN, which provides input to HVC [72]. These findings suggest that the enkephalin-positive fibers in HVC may originate from MMAN and modulate the functions of the VMP.

The other main component of the VMP, that is, RA, did not express high levels of δ-OR mRNA. However, its borders were clearly distinguishable from the surrounding arcopallium. In contrast, an earlier study from our lab has shown that the levels of OR mRNA were high in this nucleus, suggesting that these were mainly μ-ORs [also confirmed by real time PCR data, [23]]. Earlier findings [36] have demonstrated that there is no enkephalin staining in RA but it is surrounded by enkephalin-positive fibers. Taken together, these findings suggest that δ-ORs may not play a major role in modulating its functions, which may be mainly modulated by μ-ORs.

#### Anterior forebrain pathway

The anterior forebrain pathway (AFP) which is involved in song learning and context-dependent singing [32] in adulthood consists of projections connecting a subset of HVC neurons to Area X, a nucleus in the avian basal ganglia which further projects to the thalamic nucleus DLM. Projections from DLM target the pallial nucleus LMAN, which projects both to Area X and RA, forming loops [reviewed in 30, 31] within the song control system. Although strictly not a part of the AFP, we have discussed δ-OR expression in MMAN as well, given that it plays a role in song learning [72]. We observed that the core and shell regions of LMAN [73, 74] as well as MMAN express δ-ORs. Whereas levels of δ-OR mRNA were high in LMAN_shell_, these were lower in LMAN_core_, compared to the surrounding anterior nidopallium. Khurshid et al., (2009) had also demonstrated similar results with a riboprobe that detected both μ- and δ-ORs in zebra finches. However, overall levels of δ-OR mRNA were moderate, suggesting that neurons in these regions predominantly expressed μ-ORs. Our results are supported by those of Gulledge and Deviche [75], who demonstrated that δ-OR densities were lower in the LMAN of adult and young birds of both sexes, compared to the surrounding nidopallium in dark-eyed juncos (a species of seasonal songbirds).

Interestingly, Bottjer and Alexander (1995) have demonstrated that pallial regions contain a high density of enkephalin-labeled fibers and somata, with MMAN containing a slightly greater level compared to LMAN. Earlier studies have demonstrated that both MMAN and LMAN are important for vocal learning. Whereas MMAN is important for song stability in zebra finches during the sensitive period for song learning [35, 72, 76], LMAN functions in introducing variability in the songs that zebra finches produce [77]. Studies undertaken in our lab [41] have demonstrated that systemically blocking δ-ORs for a brief period during the sensorimotor phase of song learning (when juvenile zebra finches are known to learn and practice their vocalizations) alters the spectro-temporal properties of songs that treated birds sing in adulthood. Our present results therefore suggest that the δ-OR-enkephalin system may modulate vocal learning and/or vocalization in young zebra finches.

Earlier studies have demonstrated that LMAN receives projections from Area X via DLM and also projects to Area X, forming a thalamocortical basal ganglia loop [78, 79]. Whereas smaller neurons (which likely include MSNs) in Area X do not express high levels of δ-ORs (present study) or μ-ORs [23], they are highly positive for its ligand enkephalin [36]. In contrast, larger neurons in this region (cholinergic or pallidal neurons) express higher levels of δ-ORs (present study) as well as μ-ORs [23] but low levels of enkephalin [36]. Interestingly, Bottjer and Alexander (1995) have reported that a high density of enkephalin-labeled axon terminals are present throughout Area X, suggesting that δ-ORs mainly modulate pallidal neurons and perhaps, cholinergic neurons within Area X [cf. [57, 80, 81] in mammals]. In juncos, δ-OR densities are high in Area X and this area can be delineated from the surrounding MSt based on labeling intensities [75], which is not seen in zebra finches, suggesting that the δ-ORs modulate the AFP to different extents in different species of birds. Furthermore, Gulledge and Deviche (1999) have also shown that δ-OR densities are high in Area X of juvenile males compared to that in adult males, wherein levels of δ-ORs are similar in Area X and MSt. Levels of staining for enkephalin in neuronal somata present in MSt, the ‘shell’ region surrounding Area X [73, 82] are similar to those in Area X [36]. However, there appears to be a mismatch between δ-ORs and enkephalinergic fibers in MSt since this region possesses low to moderate staining for δ-ORs but the highest levels of staining for enkephalinergic fibers along its medial to ventral borders. Similar discrepancies in the expression of opioid receptors and the distribution of their ligands have been reported in pigeons [52] and rodents [83, 84]. The presence of δ-ORs specifically in Area X in young male juncos suggest that they may play a role in song learning, which may be the case in zebra finches as well [cf. [85, 86]]. Interestingly, one-day old chicks trained on a one-trial passive avoidance task demonstrated not only a significant increase in the density of δ-ORs [87] but also an increase in levels of met-enkephalin [88] in MSt. These findings suggest that δ-ORs may be involved in different aspects of learning involving the medial striatum across different species of birds.

We observed that the thalamic component of the AFP, that is, DLM, was not well-labeled for the δ-OR mRNA. However, Bottjer and Alexander (1995) had demonstrated that this region receives a dense innervation of enkephalinergic fibers and few or no positive somata. Since neuropil and small neurons in DLM demonstrate immunoreactivity for μ-ORs in zebra finches [23] and enkephalin also binds to μ-ORs [89], it is possible that its functions are modulated by μ-ORs rather than δ-ORs.

### Expression of δ-ORs in the auditory system

We also observed the expression of δ-ORs in auditory areas. High order auditory association areas NCM and CMM expressed high levels of δ-OR mRNA, followed by NIf, whereas the thalamo-recipient area Field L, thalamic nucleus Ov, midbrain nucleus MLd and the RA cup [which receives input from Field L1 and L3 (primary auditory cortex), caudal mesopallium (CM, an auditory association area), and HVC shelf [90–92]] expressed the lowest levels. Low to negligible δ-OR expression has also been observed in thalamic and midbrain areas in humans and rodents [21, 22]. The pallial auditory areas CMM and NCM are involved in auditory discrimination and in the storage of song memories [93–96], whereas NIf as well as the nucleus Avalanche (Av) link the auditory system with the circuitry for vocal control, via their direct and indirect projections to HVC. The NIf also provides a premotor drive to HVC since the firing pattern in NIf precedes HVC and inactivation of NIf in juvenile songbirds leads to degradation of plastic song to subsong [34, 97]. Our results therefore suggest that besides modulating components of the vocal motor pathway including HVC and RA, the δ-OR system may also modulate vocalizations at a premotor level via NIf.

The RA cup appears to integrate input from different parts of the auditory cortex and projects to auditory thalamic nuclei, midbrain regions [91, 98] and the ventral tegmental area [99] which provides dopaminergic input to various song control nuclei. Whereas RA cup in adult male zebra finches did not express high levels of δ-ORs, autoradiography experiments demonstrated that this area expressed high densities of δ-ORs in juncos [75].

The expression of low levels of δ-ORs in MLd (comparable to the inferior colliculus in mammals) and high levels in the surrounding ICo (nucleus intercollicularis), a part of the central gray in zebra finches [100] are remarkably similarly across zebra finches (present study), juncos [75], chick [53] and pigeons [52]. Earlier studies [36, 100] have demonstrated the presence of high levels of enkephalin-positive somata and fibers in ICo, compared to MLd in zebra finches, which is comparable to the pattern of δ-ORs expressed in these regions. Kingsbury et al. (2011) have also demonstrated the presence of the μ-OR ligand, β-endorphin in this region. Interestingly, the ICo plays a role in social interactions and mating in zebra finches [100] and stimulating this area in different species of birds elicits vocalizations [101]. Furthermore, activating the avian ICo elicits emotions including fear and aggressive displays [102]. Taken together, these findings suggest that both δ- and μ-ORs as well as other neuropeptides including Substance P, neuronal nitric oxide synthase and the catecholamines may interact to modulate the functions of ICo.

A comparison of δ-OR expression with the distribution of enkephalin-positive elements in the zebra finch brain revealed that whereas Field L, RA cup and MLd expressed low levels of δ-ORs, there were sparsely distributed enkephalin-positive somata and fibers in Field L, moderate levels of enkephalin-labeled fibers in RA cup and moderate to high levels of somata and fibers in MLd [36]. These findings are also illustrative of a mismatch between δ-ORs and their ligands as shown in other avian species [cf. 52]. The exception to generally low levels of δ-ORs in the zebra finch auditory system was provided by NCM and CMM, which were characterized by high expression of δ-ORs but sparse and uniformly distributed enkephalin-positive somata and fibers [36].

### Conclusions and future directions

Despite the fact that there are lower levels of δ-ORs compared to μ-ORs in the zebra finch brain (cf. [23]), these receptors are present across a number of cortical and subcortical structures. These findings suggest that δ-ORs may be important for modulating diverse brain functions such as cognition, spatial learning, movements and motor planning, hearing and vocalization, besides social interactions in zebra finches at different stages of development. Infusions of specific OR agonists or antagonists in various components of the song control and auditory systems, coupled with electrophysiological experiments and behavioral studies would be important to elucidate various roles of the endogenous opioid system in the zebra finch brain.

## Supporting information

S1 Fig A schematic representation of the zebra finch song control system. Vocal motor pathway (VMP; *in red*): A subset of neurons in the nidopallial nucleus HVC project to the motor nucleus RA which further projects to nXIIts (the tracheosyringeal part of the hypoglossal nerve). This nerve innervates muscles of the syrinx or vocal organ. Anterior forebrain pathway (AFP; *blue*) Another subset of HVC neurons projects to Area X of the avian basal ganglia. Area X projects to the thalamic nucleus DLM which further projects to the cortical nucleus, LMAN. Projections from LMAN form loops by innervating Area X as well as RA. Auditory pathway (*orange*): Ascending auditory information reaches the thalamic nucleus Uva which projects to HVC. Uva also projects to NIf and Av, which in turn project to HVC. Ascending auditory projections from the midbrain nucleus MLd innervate the thalamic nuclei Ov which projects to Field L. Field L sends projections to the high order auditory area NCM which is interconnected with another auditory association area, CMM [adapted from (30,34,103)]. Av, Nucleus avalanche; CMM, Caudomedial mesopallium; DLM, Dorsolateral nucleus of the medial thalamus; LMAN, Lateral magnocellular nucleus of the anterior nidopallium; MLd, Nucleus mesencephalicus lateralis, pars dorsalis; NCM, Caudomedial nidopallium; NIf, Nucleus interfaciallis nidopallii; Ov, Nucleus ovoidalis; RA, Robust nucleus of arcopallium; Uva, Nucleus uvaeformis.
(TIF)

S2 Fig Expression of δ-OR sense probe and δ-OR in the cerebellum. Negative controls were performed by staining sections with the δ-OR sense probe which demonstrates negligible label in (**A**) LMAN and (**B**) HVC. **(C)** Expression of δ-ORs is high in Purkinje cells of the cerebellum wherein several labeled neurons can be observed. Granular and molecular layers demonstrate sparsely distributed neurons which are intensely stained for the δ-OR mRNA. Scale bar, 100 μm.
(TIF)

S3 Fig The expression of δ-OR mRNA in the zebra finch striatum and pallium. (A) The MSt displays lower levels of staining compared to the surrounding nidopallium. All three pallial divisions in the anterior forebrain including the (B) hyperpallium, (C) mesopallium and (D) nidopallium express δ-OR mRNA. Levels of δ-OR expression are the highest in the nidopallium compared to other parts of the pallium. (E) Expression of δ-OR mRNA in the hippocampus (Hi2) demonstrating intensely stained neurons. (F) The dorsal intermediate arcopallium (AId) shows moderately stained large neurons which can be easily delineated from the surrounding arcopallial areas. Scale bar, 10 μm and 100 μm.
(TIF)

S4 Fig High magnification views demonstrating the expression of δ-OR in various parts of the basal ganglia and pallium. Amongst regions of the basal ganglia, (A) LSt is moderately labelled for δ-ORs, whereas pallidal cells in (B) GP are intensely label. At the level of the caudal telencephalon, neurons in (C) NCL and (D) NCM demonstrate intense label. (E) Levels of staining for δ-OR mRNA are very low in the auditory thalamo-recipient area Field L, whereas (F) the secondary auditory area CMM expressed high levels of δ-OR mRNA. The (G) RA cup, which is located in the arcopallium demonstrated low levels of label for δ-OR mRNA whereas the surrounding (H) arcopallial neurons showed intense label. Scale bar, 10 μm.
(TIF)

S1 File (XLSX)
