# Supplementary figures and images for: The expression of delta opioid receptor mRNA in adult male zebra finches (Taenopygia guttata)
Source: PLoS One. 2021 Aug 31;16(8):e0256599. doi: 10.1371/journal.pone.0256599 (PMC8407588; doi:10.1371/journal.pone.0256599)

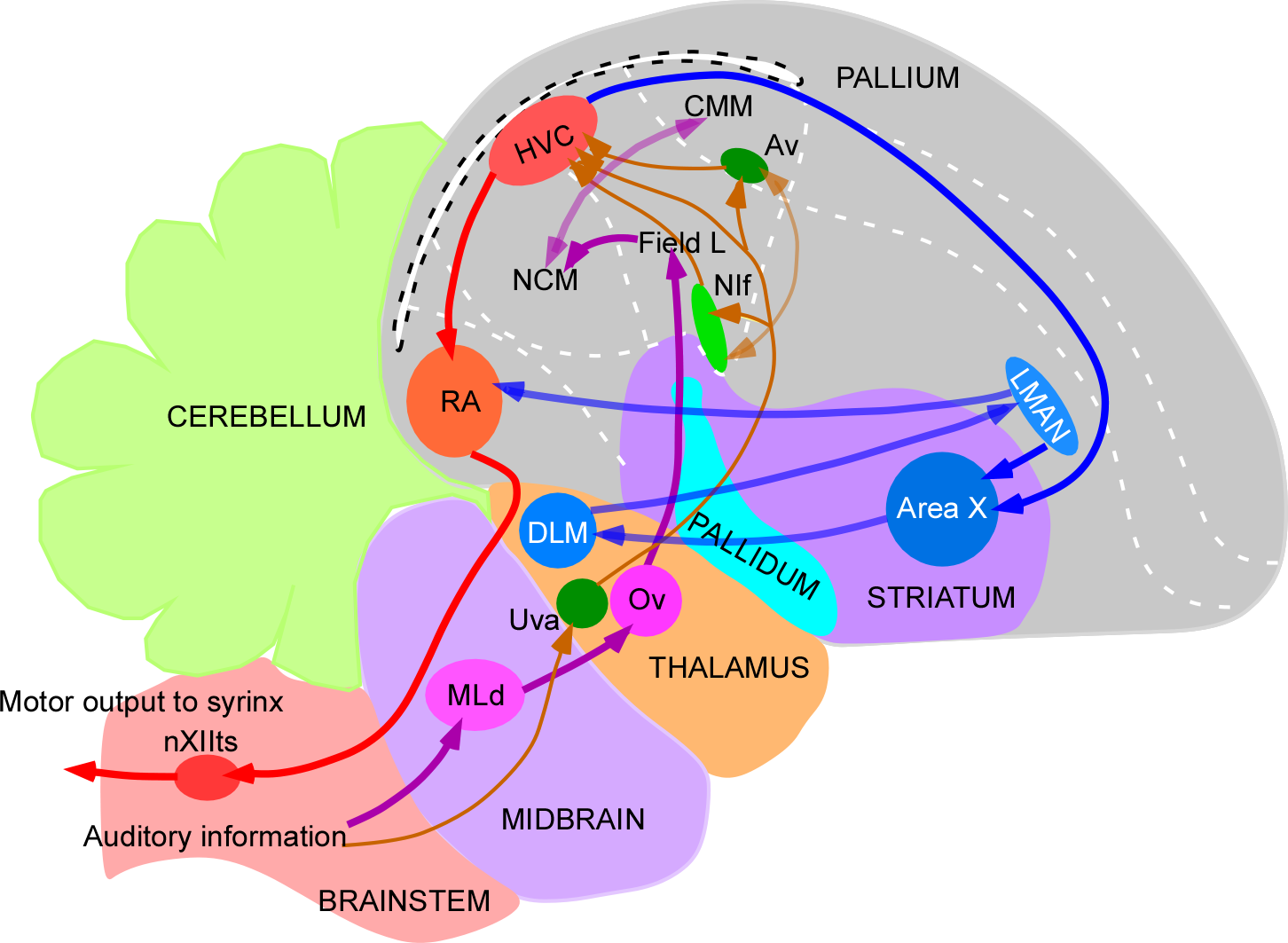

Supplement: S1 Fig — Vocal motor pathway (VMP; in red): A subset of neurons in the nidopallial nucleus HVC project to the motor nucleus RA which further projects to nXIIts (the tracheosyringeal part of the hypoglossal nerve). This nerve innervates muscles of the syrinx or vocal organ. Anterior forebrain pathway (AFP; blue) Another subset of HVC neurons projects to Area X of the avian basal ganglia. Area X projects to the thalamic nucleus DLM which further projects to the cortical nucleus, LMAN. Projections from LMAN form loops by innervating Area X as well as RA. Auditory pathway (orange): Ascending auditory information reaches the thalamic nucleus Uva which projects to HVC. Uva also projects to NIf and Av, which in turn project to HVC. Ascending auditory projections from the midbrain nucleus MLd innervate the thalamic nuclei Ov which projects to Field L. Field L sends projections to the high order auditory area NCM which is interconnected with another auditory association area, CMM [adapted from (30,34,103)]. Av, Nucleus avalanche; CMM, Caudomedial mesopallium; DLM, Dorsolateral nucleus of the medial thalamus; LMAN, Lateral magnocellular nucleus of the anterior nidopallium; MLd, Nucleus mesencephalicus lateralis, pars dorsalis; NCM, Caudomedial nidopallium; NIf, Nucleus interfaciallis nidopallii; Ov, Nucleus ovoidalis; RA, Robust nucleus of arcopallium; Uva, Nucleus uvaeformis. (TIF) [file pone.0256599.s001.tif]

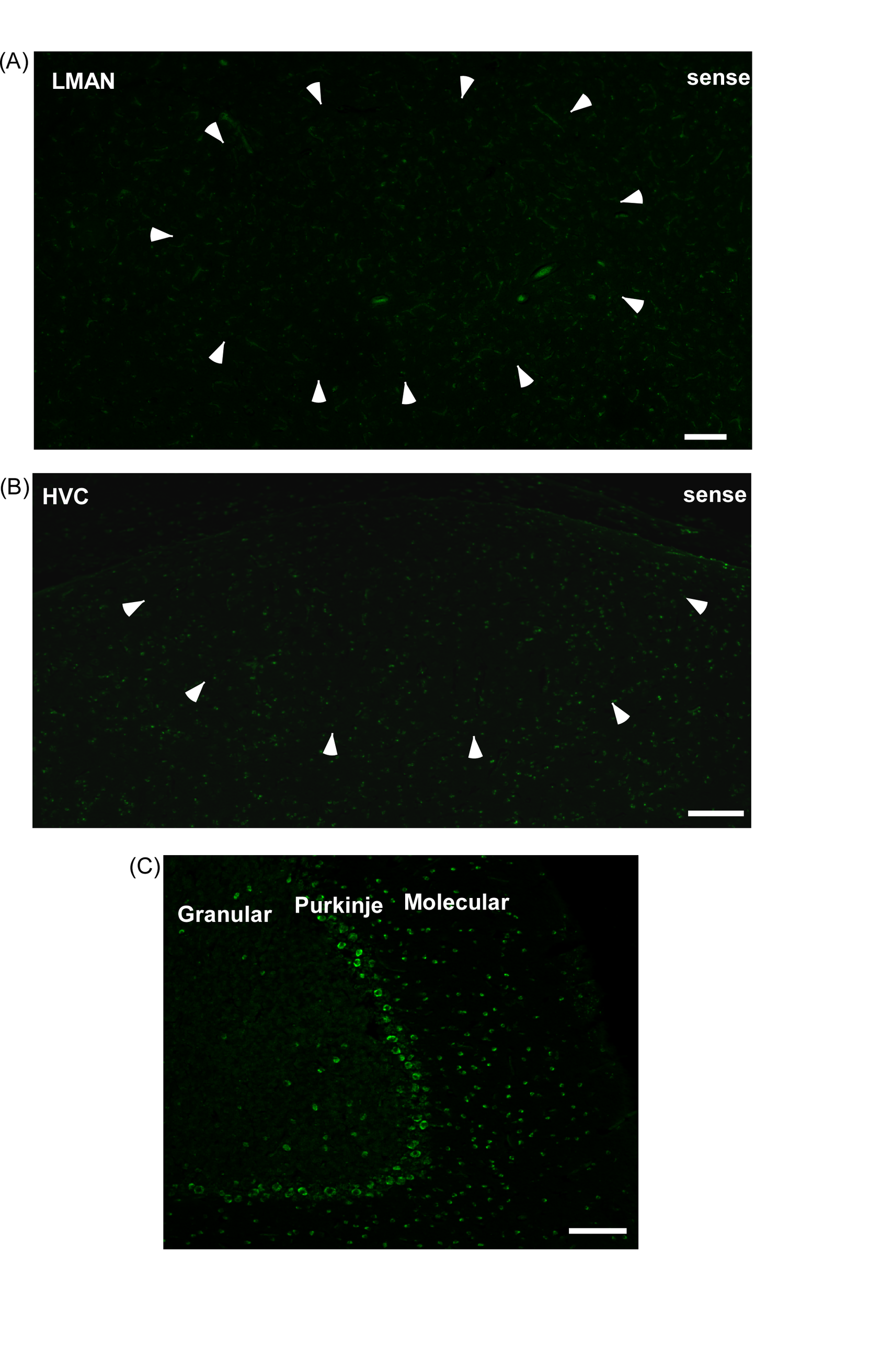

Supplement: S2 Fig — Negative controls were performed by staining sections with the δ-OR sense probe which demonstrates negligible label in (A) LMAN and (B) HVC. (C) Expression of δ-ORs is high in Purkinje cells of the cerebellum wherein several labeled neurons can be observed. Granular and molecular layers demonstrate sparsely distributed neurons which are intensely stained for the δ-OR mRNA. Scale bar, 100 μm. (TIF) [file pone.0256599.s002.tif]

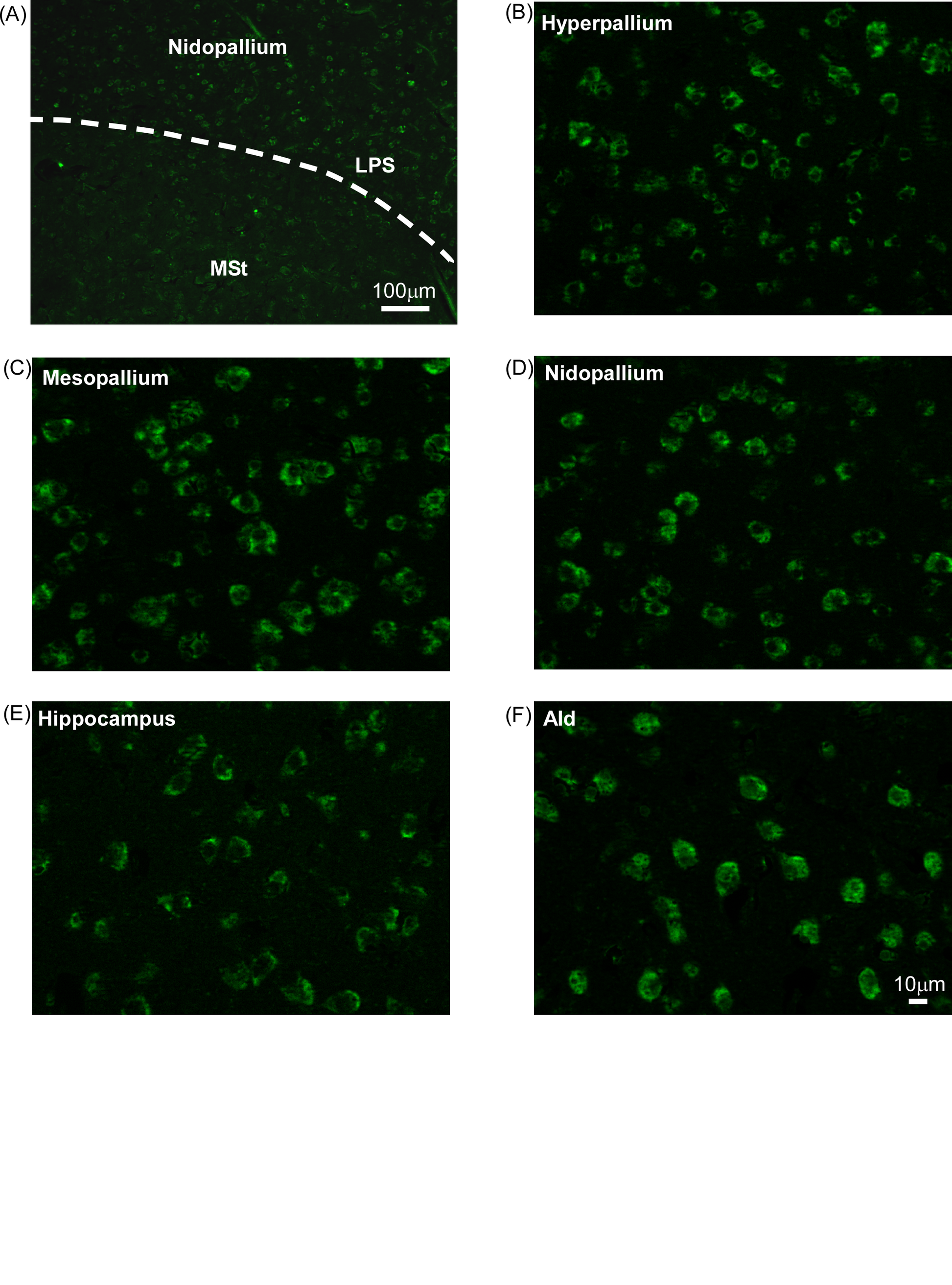

Supplement: S3 Fig — (A) The MSt displays lower levels of staining compared to the surrounding nidopallium. All three pallial divisions in the anterior forebrain including the (B) hyperpallium, (C) mesopallium and (D) nidopallium express δ-OR mRNA. Levels of δ-OR expression are the highest in the nidopallium compared to other parts of the pallium. (E) Expression of δ-OR mRNA in the hippocampus (Hi2) demonstrating intensely stained neurons. (F) The dorsal intermediate arcopallium (AId) shows moderately stained large neurons which can be easily delineated from the surrounding arcopallial areas. Scale bar, 10 μm and 100 μm. (TIF) [file pone.0256599.s003.tif]

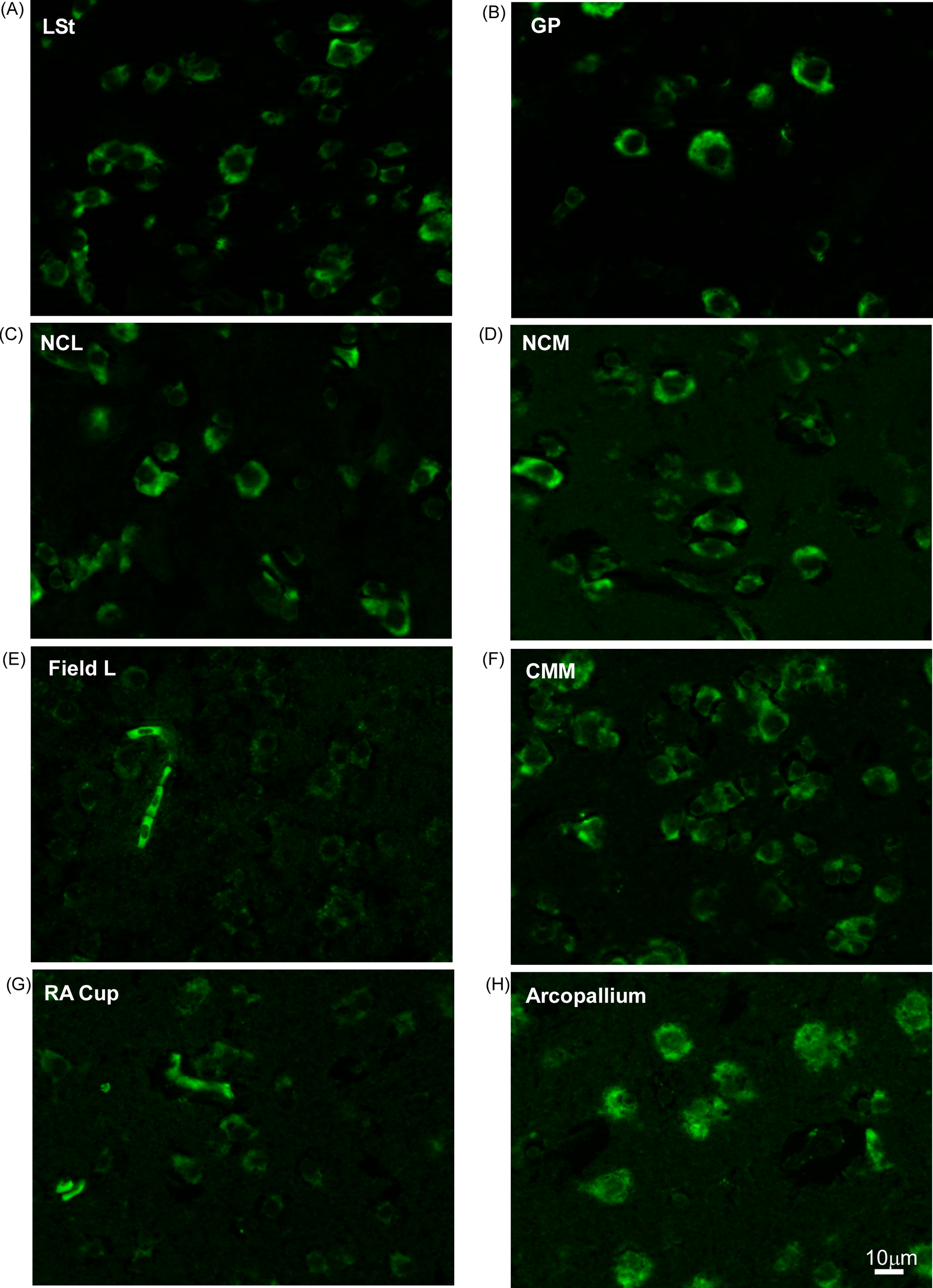

Supplement: S4 Fig — Amongst regions of the basal ganglia, (A) LSt is moderately labelled for δ-ORs, whereas pallidal cells in (B) GP are intensely label. At the level of the caudal telencephalon, neurons in (C) NCL and (D) NCM demonstrate intense label. (E) Levels of staining for δ-OR mRNA are very low in the auditory thalamo-recipient area Field L, whereas (F) the secondary auditory area CMM expressed high levels of δ-OR mRNA. The (G) RA cup, which is located in the arcopallium demonstrated low levels of label for δ-OR mRNA whereas the surrounding (H) arcopallial neurons showed intense label. Scale bar, 10 μm. (TIF) [file pone.0256599.s004.tif]
